# Joint Dual-Branch Denoising for Underwater Stereo Depth Estimation

**DOI:** 10.3390/s25227094

**Published:** 2025-11-20

**Authors:** Jingxin Zhou, Yeqi Hu, Yuan Rao, Hao Fan

**Affiliations:** College of Information Science and Engineering, Ocean University of China, Qingdao 266100, China; zhoujingxin@stu.ouc.edu.cn (J.Z.); huyeqi@stu.ouc.edu.cn (Y.H.); raoyuan@ouc.edu.cn (Y.R.)

**Keywords:** depth estimation, stereo matching, underwater, image denoising

## Abstract

Accurate depth estimation is fundamental for underwater applications such as robotics and marine exploration. However, underwater imaging suffers from severe degradation due to light attenuation, scattering, and geometric distortion, which is compounded by the scarcity of real stereo data. To address these challenges, we propose Joint Dual-Branch Denoising (JDBD), which is a plug-in framework embedded within dual-branch depth estimation networks. JDBD performs task-aware denoising via bidirectional refinement between a monocular and a stereo pathway: the monocular branch combines Adaptive White Balance and a Red Inverse Channel Prior for color correction and haze suppression, while the stereo branch applies Joint Bilateral Filtering to reduce scattering and preserve edges. Trained on the synthetic UWStereo dataset and evaluated on the real-world SQUID dataset as well as a subset of UWStereo, JDBD achieves high depth estimation accuracy and visual fidelity in underwater scenes, demonstrating robust and adaptable performance across diverse conditions.

## 1. Introduction

In underwater vision, there is an urgent demand for high-precision environmental perception techniques. As one of the most fundamental problems, depth estimation holds enormous potential to advance a broader range of underwater tasks—particularly in the field of coastal observation [1,2,3,4,5,6]. Among various sensing approaches—including multibeam/side-scan sonar and bathymetric LiDAR—stereo-based underwater depth estimation remains one of the most promising techniques due to its direct geometric formulation and high accuracy potential in shallow to mid-range waters. Sonar is robust to turbidity and long ranges but typically provides coarser spatial resolution and weak textural cues; bathymetric LiDAR performs best in clear shallow waters and requires specialized, costly hardware and careful logistics. In contrast, passive optical stereo can be built from commodity cameras, delivers centimeter-level structure with natural color, and is therefore a practical and economical choice for many diver/ROV-scale applications. Nevertheless, the effective range of passive optical stereo is constrained by turbidity and available light. Our dual-branch denoising (AWB + RCP for monocular; JBF for stereo) is explicitly designed to mitigate these failure modes while preserving the low-cost footprint of passive sensing.

Despite the remarkable progress achieved in the field of terrestrial stereo depth estimation [7,8,9,10], transferring these advances underwater remains highly challenging. Affected by wavelength-selective attenuation, backscatter, and inhomogeneous turbulence, underwater images suffer from multiple degradations, including edge blurring, hazing, and detail loss. Therefore, stereo depth estimation for multi-degraded underwater images has become one of the most challenging topics in current underwater vision [11,12].

Existing research generally follows three directions. First, there is directly transferring large pretrained terrestrial models: million-parameter models such as Foundation-Stereo [8] and MonSter [7] have demonstrated excellent generalization capabilities in adverse weather scenarios. However, when directly applied to underwater environments, they often misclassify hazy regions as objects and suffer from severe edge deviation, leading to significant performance degradation. Second, there are pre-denoising approaches prior to depth estimation. Traditional dehazing [13] and contrast enhancement [14,15] algorithms can improve visual perception but tend to sacrifice details or amplify noise; deep learning-based enhancement networks [16,17] are mostly optimized for object detection [18,19] or segmentation [20,21] tasks, which are inconsistent with the geometric fidelity requirements of depth estimation. Another often-overlooked issue is that existing methods struggle to simultaneously address various challenges in underwater depth estimation: contrast enhancement algorithms tend to intensify noise in hazy regions, while dehazing algorithms easily cause detail loss. Additionally, to avoid the high cost of acquiring real underwater stereo datasets, mainstream works [22,23] synthesize large-scale training data using terrestrial stereo datasets and underwater imaging models, and they also train monocular or stereo matching networks on this basis. However, the inherent problems of the domain gap and insufficient realism limit their practical application effects.

In summary, synthetic underwater datasets lack real degradation priors, large-model transfer ignores underwater domain differences, and image enhancement fails to simultaneously balance dehazing, edge preservation, and detail retention. These issues result in significant errors in the final depth maps within occluded regions, low-texture regions, and distant regions.

To address these challenges, we propose Joint Dual-Branch Denoising (JDBD), which is a plug-in framework embedded within a dual-branch depth estimation network. JDBD introduces targeted denoising for both monocular and stereo pathways and enables bidirectional refinement between them. The monocular branch integrates Adaptive White Balance (AWB) and Red Inverse Channel Prior (RCP) for color correction and depth-aware dehazing, while the stereo branch applies Joint Bilateral Filtering (JBF) to suppress scattering and preserve geometric structures. Through this dual-pathway interaction, JDBD generates high-fidelity, full-range underwater depth maps.

Experiments on the UWStereo [24] synthetic dataset and the SQUID [25] real-world dataset demonstrate that incorporating JDBD notably improves underwater depth estimation accuracy and visual quality, showing robust performance under diverse conditions. The main contributions of this paper are summarized as follows:

- We propose the dual-branch joint denoising framework for underwater stereo depth estimation, achieving a balanced optimization of dehazing, edge preservation, and detail retention.
- We design three lightweight modules—AWB, RCP, and JBF—to perform targeted compensation for distinct degradations in monocular and stereo pathways.
- We show that JDBD can serve as a plug-in for dual-branch depth networks, providing a transferable paradigm for the unified development of terrestrial and underwater depth estimation.

## 2. Related Work

### 2.1. Terrestrial Depth Estimation

Research on terrestrial depth estimation has evolved from traditional stereo pipelines to large-scale learning architectures that jointly model geometry and context. Early works, such as Scharstein and Szeliski [26], established the classical four-stage pipeline and evaluation benchmarks. DispNet [27] initiated the deep learning era with end-to-end disparity regression, while GC-Net [28] introduced 3D convolutions for cost aggregation. Recent models, including RAFT-Stereo [9] and IGEV-Stereo [10], refine disparity fields through recurrent updates and geometric priors, achieving top performance on KITTI [29]. MonSter [7] further integrates monocular and stereo cues in a dual-branch structure for iterative refinement. Despite their success, these models are trained and evaluated in air, and their performance degrades sharply in underwater domains due to color shift, turbidity, and refraction. This motivates us to adapt MonSter [7] as the backbone for our underwater-oriented dual-branch denoising framework.

### 2.2. Underwater Depth Estimation

Underwater depth estimation is complicated by light attenuation, backscatter, and non-uniform illumination, leading to haze and color distortion. Early studies incorporated physical imaging models. Schechner and Karpel [30] first combined polarization with stereo geometry, while Trucco and Olmos-Antillón [31] modeled light propagation to infer relative depths. Priors such as the Dark Channel [32] and the “hazy-line” prior [25] were later adapted to improve disparity consistency. With the rise of deep learning, data scarcity became a major bottleneck. Synthetic datasets such as US-Synth-20k [33] and UWStereo [24] simulate underwater conditions via rendering engines, providing valuable training resources. Recent learning-based models, e.g., UWStereoNet [11] and AQUA-DPT, incorporate degradation-aware components and Transformer architectures to handle underwater distortions. Nevertheless, these models still lag behind terrestrial counterparts in geometric accuracy. Bridging this gap requires leveraging the structural strengths of large-scale terrestrial models while tailoring them to underwater degradations—a goal pursued in our proposed JDBD framework.

### 2.3. Underwater Image Enhancement

Underwater image enhancement aims to restore color and contrast degraded by absorption and scattering. Classical approaches rely on physical models such as the Jaffe–McGlamery model [34] or polarization priors [30]. Later, statistical priors including the Dark Channel [32], UDCP [35], and Red Channel Prior [36] were developed to correct color attenuation. Data-driven methods have since achieved superior perceptual quality: UGAN [37] and UWGAN [38] adopt GAN-based mappings; Sea-thru [39] utilizes range-based physics correction; Diffusion-UIE [40] introduces cross-spectral diffusion priors for stable restoration. However, most enhancement networks target aesthetic restoration rather than geometry preservation, leading to inconsistency when used for depth estimation. In contrast, our work introduces three task-specific denoising modules—AWB, RCP, and JBF—within a dual-branch framework explicitly optimized for depth estimation fidelity.

## 3. Method

**Figure 1:**
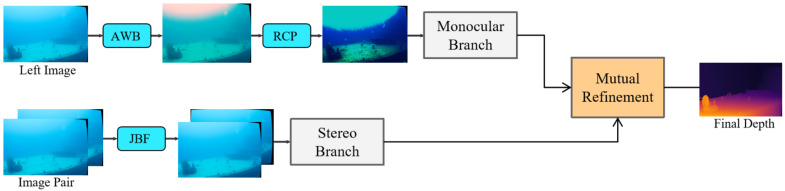
Overview of the underwater depth estimation pipeline based on stereo adaptation networks. Input stereo images are processed by a dual-branch denoising network: the monocular branch uses Adaptive White Balance (AWB) and Red Inverse Channel Prior (RCP), while the stereo branch uses Joint Bilateral Filtering (JBF). The refined images then proceed to the mutual refinement stage for depth estimation.

We propose a Joint Dual-Branch Denoising framework to address the accuracy drop of underwater stereo depth estimation caused by multiple image degradations. The architecture comprises three lightweight modules—Adaptive White Balance (AWB), Red Inverse Channel Prior (RCP), and Joint Bilateral Filtering (JBF). All three are adapted from classical image processing and prior-based restoration—gray-world white balancing, red/underwater dark-channel priors [36,41], and joint bilateral filtering [42]—but are re-parameterized and positioned for stereo depth fidelity. Concretely, AWB computes per-channel gains from a high-confidence luminance subset to expand the attenuated red band while preserving cross-view photometric consistency; RCP inverts the red channel and imposes channel-coupled transmission constraints under the underwater imaging model to suppress haze without over-correction; JBF is applied only to the stereo branch as a cross-view-guided filter that reduces scattering while keeping epipolar-consistent edges. Integrated via the dual-branch design (AWB→RCP on the monocular path; JBF on the stereo path), these modules provide complementary, task-aware denoising before the mutual refinement stage, as illustrated in Figure 1.

### 3.1. Targeted Denoising for the Monocular Branch

The preprocessing for the monocular branch follows an AWB→RCP order designed for task adaptation. Adaptive White Balance (AWB) is first applied as spatially invariant per-channel gains to neutralize color cast and increase local contrast, maximizing the retention of texture details and faint distant cues.This step inevitably accentuates backscatter in turbid regions. RCP is subsequently applied to compensate range-dependent attenuation and suppress backscatter, producing an input to the monocular branch that preserves details while remaining visually clear. Within the dual-branch architecture, the monocular branch leverages this preprocessed image to capture rich textures and distant scene layout, whereas the stereo branch supplies precise depth for fine structures via disparity.

#### 3.1.1. Notation and Windows

Let I(x,λ)∈[0,1] be the observed intensity at pixel *x* and channel λ∈{R,G,B}. We denote the image domain by $\Omega_{\mathrm{img}}$. Let J(x,λ) denote the scene radiance (haze-suppressed image), $B_{\lambda ,\infty }$ the background light, and t(x,λ)=exp[−c(λ)d(x)] the transmission, where c(λ) is the attenuation coefficient and d(x) the scene depth. For any pixel *x*, Ω(x) denotes a square spatial window of radius *r* (size 2r+1). For AWB, the *k*-th color-temperature bin is the intra-frame pixel set $W_{k}\subset \Omega_{\mathrm{img}}$ with cardinality $N_{k}=\left|W_{k}\right|$.

#### 3.1.2. Adaptive White Balance (AWB)

Define channel sums and means over $W_{k}$:

(1) $$S_{\lambda }\left(k\right)=\sum_{i\in W_{k}}I\left(i,\lambda \right),\;\lambda \in \left\{R,G,B\right\},$$

(2) $${\bar{I}}_{\lambda }\left(k\right)=\frac{S_{\lambda }\left(k\right)}{N_{k}},\;\lambda \in \left\{R,G,B\right\}.$$

Using *G* as the neutral reference yields per-bin gains

(3) $$g_{R}\left(k\right)=\frac{{\bar{I}}_{G}\left(k\right)}{{\bar{I}}_{R}\left(k\right)},\;g_{G}\left(k\right)=1,\;g_{B}\left(k\right)=\frac{{\bar{I}}_{G}\left(k\right)}{{\bar{I}}_{B}\left(k\right)}.$$

and the corrected output for pixels $x\in W_{k}$:

(4) $$I_{\mathrm{out}}\left(x,\lambda \right)=I_{\mathrm{in}}\left(x,\lambda \right)\cdot g_{\lambda }\left(k\right).$$

We compute $W_{k}$ from a luminance-confident subset intersected with the *k*-th CCT bin, which stabilizes gray-world estimation under wavelength-selective attenuation.

#### 3.1.3. Red Inverse Channel Prior (RCP)

The classical dark channel prior (DCP) assumes that in a haze-free patch, at least one channel is nearly zero:

(5) $$J^{\mathrm{DCP}}\left(x\right)=\min_{\lambda \in \{R,G,B\}}\;\min_{y\in \Omega (x)}I\left(y,\lambda \right)\;\approx 0.$$

Underwater, strong red attenuation violates this assumption; we therefore invert the red channel before the min operator:

(6) $$J^{\mathrm{RCP}}\left(x\right)=\min\left\{\min_{y\in \Omega (x)}\left(1-I(y,R)\right),\;\min_{y\in \Omega (x)}I\left(y,G\right),\;\min_{y\in \Omega (x)}I\left(y,B\right)\right\}.$$

##### Underwater Image Formation and Transmission

The formation model is

(7) $$I\left(x,\lambda \right)=J\left(x,\lambda \right)\;t\left(x,\lambda \right)+B_{\lambda ,\infty }\;\left(1-t(x,\lambda )\right),\;t\left(x,\lambda \right)=\exp\left[-c\left(\lambda \right)\;d\left(x\right)\right].$$

We estimate $B_{\lambda ,\infty }$ from a red score

(8) $$S_{R}\left(x\right)=I\left(x,R\right)-\max\;\left(I(x,G),I(x,B)\right),$$

and choose a small set (e.g., top 0.1%) of high-$S_{R}$ pixels as candidates. Assuming locally constant $B_{\lambda ,\infty }$ and invoking the dark-channel approximation, the coarse transmission is

(9) $$\hat{t}\left(x,\lambda \right)=1-\min_{y\in \Omega (x)}\frac{I(y,\lambda )}{B_{\lambda ,\infty }},\;\hat{t}\left(x,R\right)=1-\min_{y\in \Omega (x)}\frac{1-I(y,R)}{\;1-B_{R,\infty }\;},$$

where the red-channel expression follows the inversion in (6).

##### Spectral Coupling

Using the scattering–attenuation relation

(10) $$B_{\lambda ,\infty }\;\propto \;\frac{b(\lambda )}{c(\lambda )},$$

we obtain

(11) $$\frac{c(G)}{c(R)}=\frac{b\left(G\right)\;B_{R,\infty }}{b\left(R\right)\;B_{G,\infty }},\;\frac{c(B)}{c(R)}=\frac{b\left(B\right)\;B_{R,\infty }}{b\left(R\right)\;B_{B,\infty }},$$

and thus channel-coupled transmissions

(12) $$\hat{t}\left(x,G\right)={\left(\hat{t}\left(x,R\right)\right)}^{c(G)/c(R)},\;\hat{t}\left(x,B\right)={\left(\hat{t}\left(x,R\right)\right)}^{c(B)/c(R)}.$$

Finally, the restored radiance is

(13) $$J\left(x,\lambda \right)=\frac{I\left(x,\lambda \right)-B_{\lambda ,\infty }}{\max\;\left(\hat{t}\left(x,\lambda \right),\;t_{\min}\right)}+B_{\lambda ,\infty }.$$

with a small $t_{\min}$ to avoid amplification.

### 3.2. Targeted Denoising for the Stereo Branch

Before disparity estimation, we apply bilateral filtering to each view to suppress scattering while preserving edges. For a pixel *p* in the left image and a spatial window Ω(p),

(14) $$J_{l}\left(p\right)=\frac{1}{W_{p}}\sum_{q\in \Omega (p)}G_{\sigma_{s}}\;\left(∥p-q∥\right)\;G_{\sigma_{r}}\;\left(|I_{l}\left(p\right)-I_{l}\left(q\right)|\right)\;I_{l}\left(q\right),$$

(15) $$W_{p}=\sum_{q\in \Omega (p)}G_{\sigma_{s}}\;\left(∥p-q∥\right)\;G_{\sigma_{r}}\;\left(|I_{l}\left(p\right)-I_{l}\left(q\right)|\right),$$

where $G_{\sigma_{s}}\left(∥p-q∥\right)={\exp(-∥p-q∥}^{2}/\left(2\sigma_{s}^{2}\right))$ and $G_{\sigma_{r}}(|I\left(p\right)-I\left(q\right)|)=\exp\left(-{\left(I\left(p\right)-I\left(q\right)\right)}^{2}/\left(2\sigma_{r}^{2}\right)\right)$. When the guidance image differs (e.g., the right view guides the left),

(16) $$J_{l}\left(p\right)=\frac{\sum_{q\in \Omega (p)}G_{\sigma_{s}}(∥p-q∥)\;G_{\sigma_{r}}\;\left(|I_{l}\left(p\right)-I_{r}\left(W\left(q\right)\right)|\right)\;I_{l}\left(q\right)}{\sum_{q\in \Omega (p)}G_{\sigma_{s}}(∥p-q∥)\;G_{\sigma_{r}}\;\left(|I_{l}\left(p\right)-I_{r}\left(W\left(q\right)\right)|\right)}.$$

Here, W(q) maps a left-image pixel *q* to its guided correspondence in the right image; for rectified pairs, $W\left(q\right)=(q_{x}-d\left(q\right),\;q_{y})$ using the current disparity *d*.

The JBF suppresses scattering-induced blur and uneven illumination while maintaining geometric and photometric consistency between the stereo pair. By performing spatially and photometrically weighted filtering, it produces edge-preserving, disparity-consistent images that strengthen the reliability and stability of stereo correspondence estimation, particularly under turbid or low-contrast underwater conditions.

### 3.3. Mutual Refinement

Following the mutual refinement framework of MonSter [7], our network establishes an iterative coupling between the denoised monocular and stereo branches. After branch-wise denoising (AWB and RCP for the monocular path, and JBF for the stereo path; see Figure 1), the two branches exchange complementary cues to achieve consistent, noise-suppressed depth estimation. The refinement consists of three essential stages: global alignment, alternating update, and weighted supervision.

#### 3.3.1. Global Alignment

The relative monocular depth is converted into disparity and coarsely aligned with the stereo domain through a global scale–shift pair $\left(s_{G},t_{G}\right)$ estimated on reliable pixels Ω:

(17) $$\left(s_{G},t_{G}\right)=\arg\min_{s,t}\sum_{i\in \Omega }{\left(s\;D_{M}\left(i\right)+t-D_{S}\left(i\right)\right)}^{2},$$

(18) $${\tilde{D}}_{M}=s_{G}D_{M}+t_{G}.$$

This step provides a unified depth scale, enabling the two branches to operate within the same geometric domain.

#### 3.3.2. Alternating Update

The refinement alternates between the monocular and stereo branches for $N_{2}$ rounds after $N_{1}$ initial stereo-only iterations. Each update stage integrates cross-branch cues through learned refinement operators $\Phi_{M}$ and $\Phi_{S}$:

(19) $$D_{M}^{\left(i+1\right)}=\Phi_{M}\left({\tilde{D}}_{M}^{\left(i\right)},\;D_{S}^{\left(i\right)}\right),$$

(20) $$D_{S}^{\left(i+1\right)}=\Phi_{S}\left(D_{S}^{\left(i\right)},\;{\tilde{D}}_{M}^{\left(i\right)}\right),$$

where $i=0,\ldots ,N_{2}-1$. The preceding denoising modules ensure that each branch provides stable, low-noise structural priors, allowing the iterative updates to jointly enhance fine-scale consistency and suppress scattering-related artifacts. This cooperative process tightly couples the dual-branch denoising and depth estimation, enabling the progressive fusion of geometric and photometric information.

#### 3.3.3. Loss Function

The network is trained with L1 supervision across all iterations, using exponentially decayed weights to emphasize later refinements. The total loss combines the stereo branch loss $L_{Stereo}$ and monocular branch loss $L_{Mono}$ as follows:

(21) $$L=L_{Stereo}+L_{Mono},$$

(22) $$L_{Stereo}=\sum_{i=0}^{N_{1}-1}\gamma^{N_{1}+N_{2}-i}∥D_{S}^{i}-d_{gt}{∥}_{1}+\sum_{i=N_{1}}^{N_{1}+N_{2}-1}\gamma^{N_{1}+N_{2}-i}∥D_{S}^{i-N_{1}}-d_{gt}{∥}_{1},$$

(23) $$L_{Mono}=\sum_{i=N_{1}}^{N_{1}+N_{2}-1}\gamma^{N_{1}+N_{2}-i}∥D_{M}^{i-N_{1}}-d_{gt}{∥}_{1},$$

where γ=0.9 is the exponential decay coefficient. After $N_{2}$ rounds, the refined stereo disparity $D_{S}^{\left(N_{2}\right)}$ serves as the final output.

This dual-branch refinement effectively integrates denoising and depth estimation: the monocular path provides dehazed and spectrally corrected priors, while the stereo path enforces geometric accuracy. Through iterative cross-guidance, both branches converge toward a unified, noise-robust underwater depth representation.

## 4. Experiments

### 4.1. Datasets and Backbone Network

We conduct experiments on both synthetic and real underwater datasets to evaluate the generalization of JDBD. The synthetic UWStereo dataset [24] is used for training, and the real SQUID dataset [25] as well as a subset of UWStereo [24] is used for evaluation.

UWStereo [24] contains 29,568 stereo pairs with dense disparity annotations. It covers various underwater scenes such as corals, ships, and industrial structures, and it is generated with Unreal Engine 5 to simulate diverse cameras, lighting, and water conditions, ensuring data diversity and realism.

SQUID [25] is a real-world underwater stereo dataset including 57 image pairs captured at four sites in Israel and the Caribbean with a resolution of 2700 × 1700. The stereo rig uses two Nikon D810 cameras (Nikon Corporation, Tokyo, Japan) with AF-S NIKKOR 35 mm f/1.8 G ED lenses in Hugyfot housings with a dome port, which are mounted on a rigid bar. At each site, 20–30 checkerboard images were acquired, and the system was calibrated with the MATLAB (R2017) Stereo Calibration Toolbox; lens distortion was corrected. Dense correspondences were generated with bidirectional EpicFlow and filtered using an end-point-error threshold (<5 px), providing accurate geometry together with challenging real variations in water clarity and illumination. For fairness, all methods use the same calibration and rectified image pairs throughout training and evaluation to avoid bias from re-calibration.

For depth estimation, we adopt MonSter [7] as the backbone and embed our proposed dual-branch denoising modules. These modules transform degraded underwater inputs into denoised counterparts suitable for depth inference. We compare JDBD with seven representative stereo depth estimation networks: Gwc-Net [43], PsmNet [44], COEX [45], SAN [46], RAFT-Stereo [9], Selective-Stereo [10], and MonSter [7]. As an unsupervised method, SAN is evaluated by directly loading the authors’ pretrained underwater weights without any training or fine-tuning on UWStereo. For all other stereo networks (Gwc-Net, PSMNet, COEX, RAFT-Stereo, Selective-Stereo, and MonSter), we load official pretrained checkpoints and fine-tune them on UWStereo under the same input resolution and preprocessing. Our JDBD-enhanced MonSter follows the same fine-tuning protocol.

To assess whether generic enhancement can replace task-specific denoising, we evaluate a two-stage pipeline that first enhances each stereo view independently and then performs depth estimation. Four representative underwater enhancement networks—CLUIE-Net [47,48], NU2-Net [49], GHS-UIR [50], and HCLR-Net [51]—are used for preprocessing. Because these models are trained on lower-resolution inputs, SQUID images are downscaled to each model’s native resolution for enhancement and then resampled back to 2700 × 1700 for stereo matching. For fairness, we do not retrain the enhancement networks; all subsequent photometric normalization and evaluation steps are kept identical to our default pipeline. The left/right images are processed with the same model and parameters to preserve epipolar consistency. This setup isolates the net effect of generic enhancement on geometry-sensitive stereo correspondence.

### 4.2. Training Setup and Evaluation Metrics

#### 4.2.1. Training Setup

We train the proposed JDBD on the synthetic UWStereo dataset [24], and evaluate it on the real-world SQUID dataset [25] as well as on a subset of UWStereo [24], in order to assess both cross-domain generalization and synthetic-domain consistency. The network is implemented in PyTorch and trained using NVIDIA RTX 4090 GPUs. We employ the AdamW optimizer [52] with gradient clipping in [−1,1], following the baseline practice. A one-cycle learning rate schedule is used with a peak learning rate of $2\times 10^{-4}$. Training is conducted for 4000 steps with a batch size of 6 to obtain a pretrained model adapted to underwater conditions. We intentionally refrain from introducing underwater-specific augmentation during training beyond the baseline practice so as to isolate the contribution of JDBD itself. Instead, we evaluate robustness with synthetic turbidity and illumination only at test time, ensuring that improvements stem from our denoising design rather than from task-specific augmentation.

#### 4.2.2. Environment and Computational Cost

The implementation uses Python 3.9 and PyTorch 2.5.0. All experiments are run on a single NVIDIA RTX 4090 (24 GB). At the SQUID test resolution (2700 × 1700), end-to-end inference of JDBD requires approximately 4.6 GB of GPU memory and introduces only a small overhead compared with MonSter alone, reflecting the lightweight design of the denoising branch.

#### 4.2.3. Evaluation Metrics

For quantitative evaluation, we adopt two commonly used disparity metrics: end-point error (EPE) and D1. EPE measures the average pixel-wise disparity error between the prediction and the ground truth, while D1 is the percentage of pixels whose disparity error exceeds 3 px or 5% of ground-truth disparity. Both metrics are reported in lower-better form, reflecting higher accuracy in depth prediction.

### 4.3. Quantitative Results

Next, we compare with the stereo matching networks. Table 1 reports results on a subset of the UWStereo dataset [24]. Table 2 reports results on the real SQUID dataset [25], and across seven representative stereo depth estimation networks—Gwc-Net [43], SAN [46], PsmNet [44], COEX [45], RAFT-Stereo [9], Selective-Stereo [10], and MonSter [7]—JDBD achieves consistently lower EPE and D1 values on both datasets.

**Figure 2:**
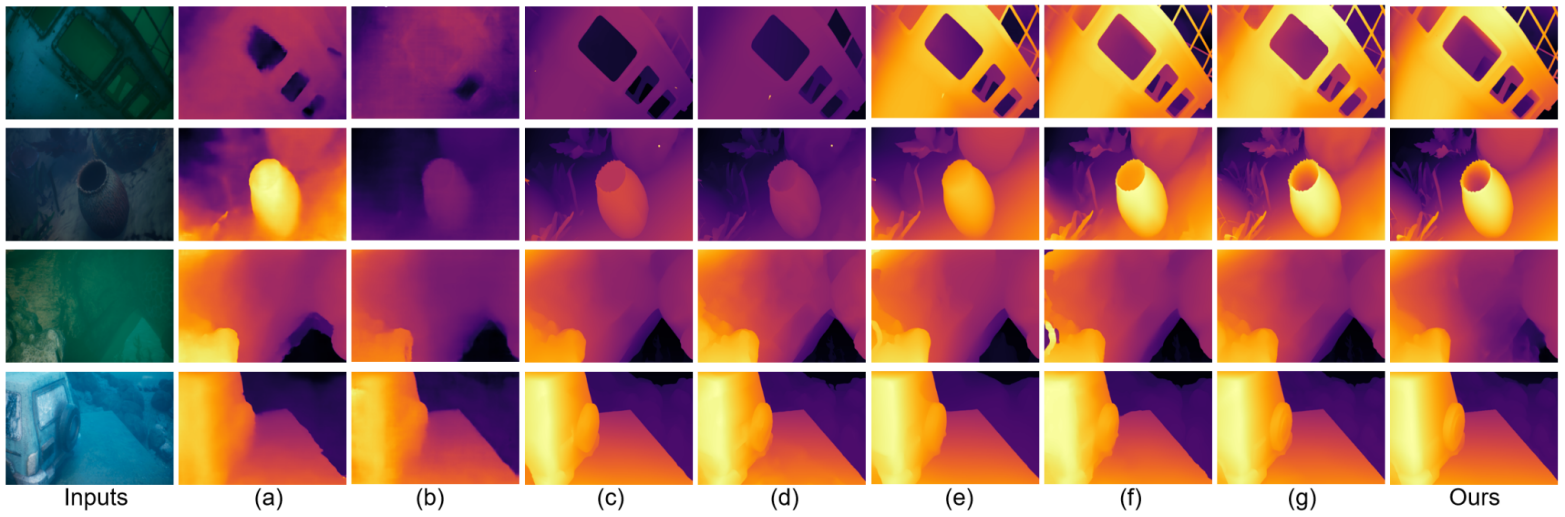
Qualitative comparison on UWStereo [24]. Rows: ship, coral, industry, default. (**a**–**g**): GwcNet [43], SAN [46] PsmNet [44], COEX [45], RAFT-Stereo [9], Selective-Stereo [10], MonSter [10], JDBD (Ours). JDBD shows clearer depths with preserved details and sharper edges.

**Figure 3:**
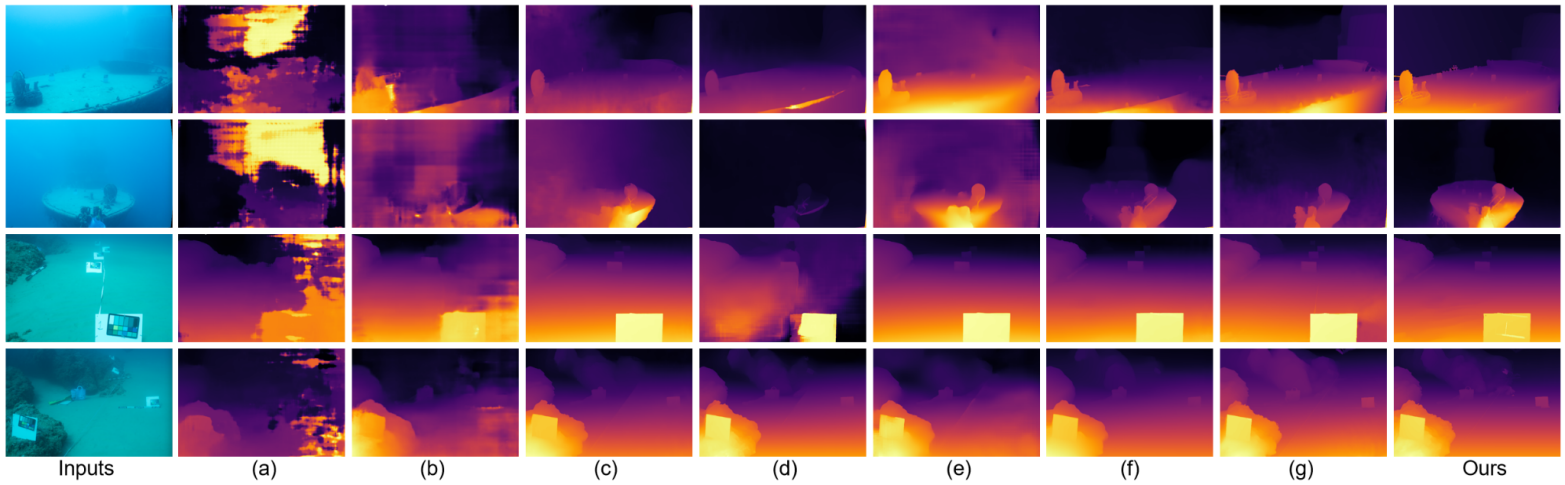
Depth estimation results on different underwater scenes from the SQUID dataset [25]. (**a**–**g**) correspond to the results of GwcNet [43], SAN [46], PsmNet [44], COEX [45], RAFT-Stereo [9], Selective-Stereo [10], and MonSter [7], respectively.

Relative to the MonSter baseline [7], JDBD reduces error by 31.7% (EPE) and 55.7% (D1) on SQUID [25] and by 33.8% (EPE) and 42.3% (D1) on the UWStereo subset. Qualitative comparisons in Figure 2 and Figure 3 show clearer object boundaries and fewer scattering artifacts, indicating improved depth fidelity. These results also suggest that models pretrained on terrestrial datasets (e.g., KITTI [29]), even after fine-tuning on UWStereo [24], can retain a noticeable domain gap on real underwater data, motivating dedicated underwater denoising and adaptation.

Next, we compare with enhancement-based pipelines. We further compare JDBD with a two-stage strategy that first enhances underwater images and then performs stereo matching. Specifically, four representative enhancement networks—CLUIE-Net [47,48], NU2-Net [49], GHS-UIR [50], and HCLR-Net [51]—are applied as preprocessing on SQUID [25], after which the enhanced images are fed into MonSter [7] and RAFT-Stereo [9]. As shown in Table 3, although these pipelines improve the visual appearance, they generally produce higher disparity errors than JDBD due to compromised geometric consistency, which is crucial for stereo correspondence. By contrast, JDBD’s dual-branch design couples depth-aware dehazing in the monocular branch with geometry-preserving cues in the stereo branch, yielding higher overall accuracy and stable performance across both synthetic and real underwater conditions.These findings indicate that simply inserting an image enhancement stage before stereo matching does not necessarily lead to better depth estimation, and that our task-specific dual-branch denoising design is more effective and geometry-consistent, even when strong backbone models such as MonSter [7] are used.

### 4.4. Qualitative Results

#### 4.4.1. Visualization on the UWStereo Dataset [24]

Figure 2 presents qualitative comparisons among seven stereo depth estimation networks on four representative UWStereo [24] scenes: coral, default, industry, and ship. Methods pretrained on terrestrial datasets (e.g., KITTI [29]) exhibit significant haze-like artifacts and detail loss when transferred to underwater domains. GwcNet [43], PsmNet [44], and COEX [45] tend to oversmooth or completely ignore distant structures, resulting in severe texture omission. As an underwater unsupervised approach, SAN [46] reduces some scattering but is strongly affected by haze/backscatter, exhibiting severe degradation in far-range regions (faded distant textures and unstable edges), which is consistent with its quantitative results. RAFT-Stereo [9] and Selective-Stereo [10] alleviate some scattering effects but still suffer from blurred boundaries and missing fine structures. MonSter [7] performs relatively better, yet mild haziness and edge softness persist. In contrast, JDBD produces the clearest depth maps with well-preserved distant textures, reduced scattering artifacts, and sharper edges, effectively recovering geometry even in low-contrast and turbid regions. These observations demonstrate that JDBD provides visually cleaner and more detailed-complete depth estimation across diverse underwater scenes.

#### 4.4.2. Visualization on the SQUID Dataset [25]

Figure 3 shows depth estimation results on the real SQUID dataset [25], including shipwreck and rock scenes. Similar to the UWStereo [24] results, JDBD achieves a favorable balance among dehazing, detail preservation, and edge clarity. Compared with other networks, its depth maps exhibit higher contrast in distant areas and improved continuity around object boundaries while effectively suppressing noise and scattering haze. In line with its unsupervised nature, SAN [46] shows noticeable residual haze and unstable edges, which corroborates the quantitative gap on SQUID [25]. This consistent improvement across synthetic and real datasets indicates the strong robustness and adaptability of JDBD in real underwater conditions.

#### 4.4.3. Visualization with Enhancement-Based Pipelines

**Figure 4:**
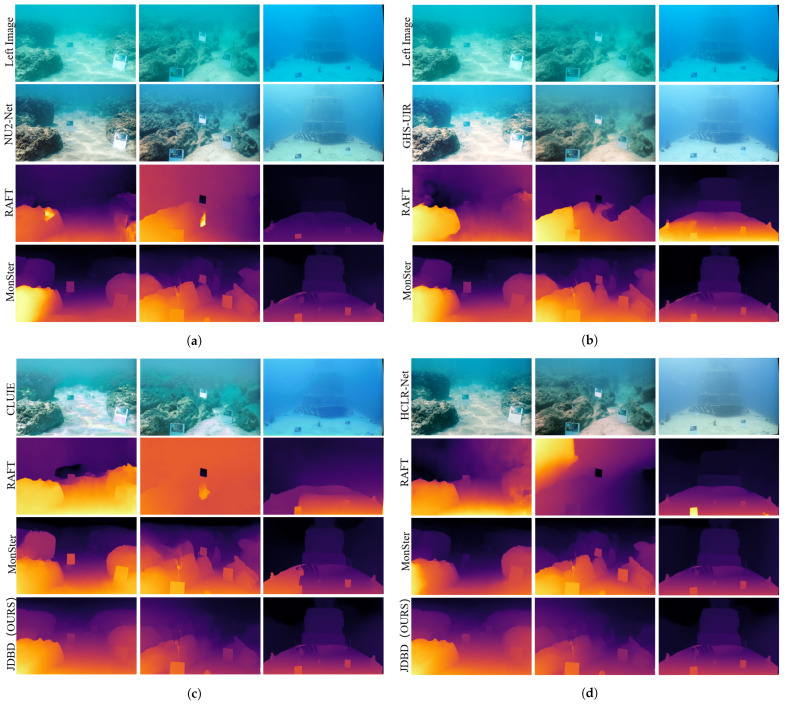
Depth estimation results on different underwater scenes from the SQUID dataset [25]. (**a**–**d**) correspond to the four underwater image enhancement networks NU2-Net [49], GHS-UIR [50], CLUIE-Net [47,48] and HCLR-Net [51], respectively.

For reference, Figure 4 further compares JDBD with pipelines that apply underwater image enhancement before stereo matching. Enhanced images generated by CLUIE-Net [47,48], NU2-Net [49], GHS-UIR [50], and HCLR-Net [51] are fed into RAFT-Stereo [9] and MonSter [7], which are recognized for strong generalization performance. While enhancement improves overall appearance, it introduces geometric inconsistencies—particularly in distant regions and fine object edges—leading to unstable disparity predictions. In contrast, JDBD integrates denoising and stereo correspondence within a unified framework, preserving both visual clarity and structural accuracy. As a result, JDBD achieves the most visually consistent and geometrically reliable depth maps across both synthetic and real underwater scenarios.

### 4.5. Performance on Ill-Posed Regions

#### 4.5.1. Robustness in Real Underwater Scenes

Ill-posed regions are common in real underwater scenes due to scattering, attenuation, and occlusions, making reliable depth estimation significantly more challenging than in terrestrial environments. To assess the robustness of JDBD in such cases, we conduct qualitative visualization on the real SQUID dataset [25], which contains naturally degraded underwater scenes characterized by hazy water, texture-missing surfaces, and edge-blurred boundaries. Two representative scenarios—coral reefs and shipwrecks—are selected for visualization.

**Figure 5:**
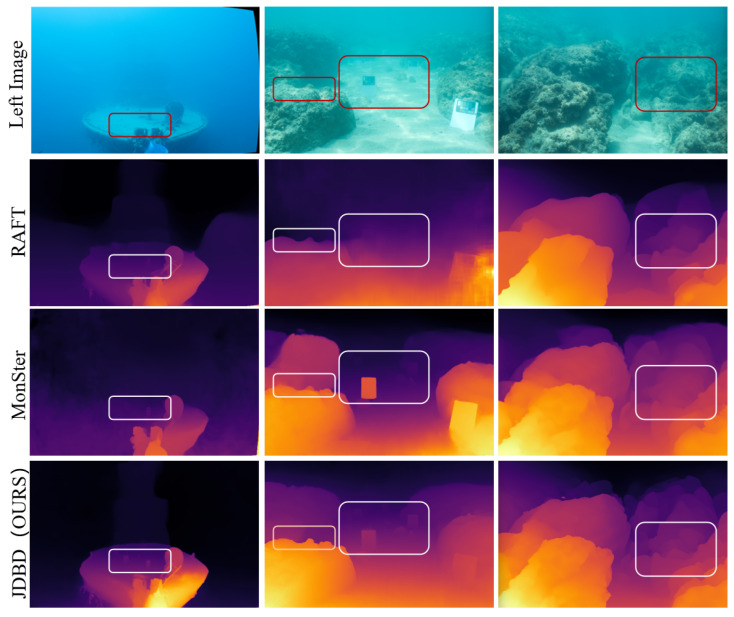
Zero-shot generalization comparison: all models are trained on the UWStereo dataset [25] and tested directly on the SQUID dataset [24]. Compared to the baseline models MonSter [7] and RAFT-Stereo [9], our proposed JDBD method shows significant performance improvement in challenging regions such as hazy regions, blurred details, fine structures, and distant objects. The boxes correspond to the challenging regions.

As shown in Figure 5, both RAFT-Stereo [9] and MonSter [7] fail to recover accurate structures in these ill-posed areas, even when their inputs are preprocessed by underwater image enhancement networks. RAFT-Stereo [9] loses most fine textures on the shipboard color checker and rope details while also exhibiting edge drift and severe haze-induced blurring. MonSter [7] alleviates part of the scattering effect but still suffers from blurred contours and significant detail loss around reefs and hull edges. In contrast, JDBD effectively balances dehazing, detail preservation, and edge sharpness. It successfully restores distant textures under heavy haze, maintains consistent depth around occluded rock boundaries, and preserves the intricate structures of ropes and color charts on ship surfaces. These results demonstrate that JDBD achieves higher visual consistency and geometric reliability in complex underwater conditions, particularly within haze-dominated, low-texture, and edge-ambiguous regions.

#### 4.5.2. Robustness to Turbidity and Illumination

We synthesize controlled degradations on SQUID [25] using a simple, physics-consistent procedure: per-channel exponential attenuation following the Beer–Lambert model with ambient-light blending, a small Gaussian blur to mimic forward scattering, and ambient estimated from the top 0.5% brightest pixels. Three turbidity levels are used (mild/medium/heavy with $\beta_{\mathrm{base}}=0.25/0.40/0.80$ and blur σ=0.5/1.0/2.0); illumination is varied by gamma and slight contrast changes (low/normal/high-key with γ=2.4/1.2/0.8 and c=0.95/1.00/1.03). To preserve epipolar consistency, the exact same degradations are applied to the left and right views of each stereo pair. For ambient-light estimation, a single value is computed per pair and shared across views, avoiding cross-view bias.

**Figure 6:**
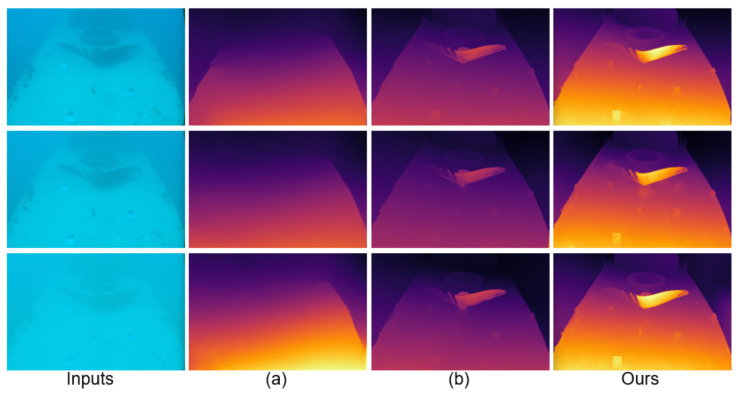
Turbidity gradient on SQUID [25]: from top to bottom, mild, medium, heavy. Columns: (**a**) RAFT-Stereo [9], (**b**) MonSter [7], JDBD+MonSter (Ours). The same degradations applied to both views.

**Figure 7:**
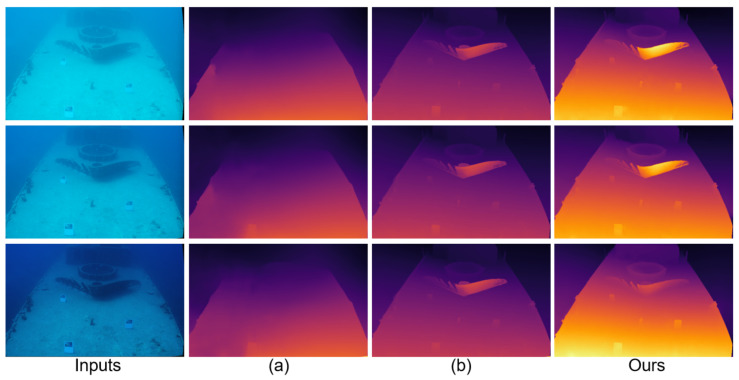
Illumination gradient on SQUID [25]: from top to bottom, high-key, normal, low-light. Columns: (**a**) RAFT-Stereo [9], (**b**) MonSter [7], JDBD (Ours). The same settings applied across all methods.

As turbidity increases from mild to heavy, the three models show clear and consistent trends. RAFT-Stereo [9] rapidly deteriorates: it already loses color-chart and rope details under mild turbidity, and it exhibits pronounced edge drift and haze-induced blurring at medium and heavy levels. MonSter remains acceptable in mild turbidity but gradually loses distant structures and fine textures as turbidity grows with visible contour smoothing and mismatch near occlusions. JDBD is the most stable across all levels, preserving most fine details and edge sharpness and showing the smallest degradation in EPE and D1. Under low-light variants, the ranking is unchanged: RAFT-Stereo [9] suffers the largest drop, MonSter [7] degrades moderately, and JDBD remains robust with only minor performance loss. Qualitative examples and quantitative summaries are presented in Figure 6 and Figure 7, Table 4 and Table 5.

### 4.6. Ablation Study

#### 4.6.1. Quantitative Analysis

To examine the contribution of each component in the proposed Joint Dual-Branch Denoising (JDBD) framework, we conduct ablation experiments using UWStereo [24] for training and the real SQUID dataset [25] for evaluation. Following the MonSter [7] backbone, the baseline model trained on mixed data shows the lowest accuracy. As shown in Table 6, the progressive introduction of the three denoising modules—Adaptive White Balance (AWB), Red Inverse Channel Prior (RCP), and Joint Bilateral Filtering (JBF)—leads to consistent performance gains. The complete JDBD reduces the average EPE by 31.7% (from 1.83 to 1.25) and the D1 error by 55.7% (from 6.72 to 2.98), confirming the effectiveness of the proposed design.

#### 4.6.2. Qualitative Analysis

**Figure 8:**
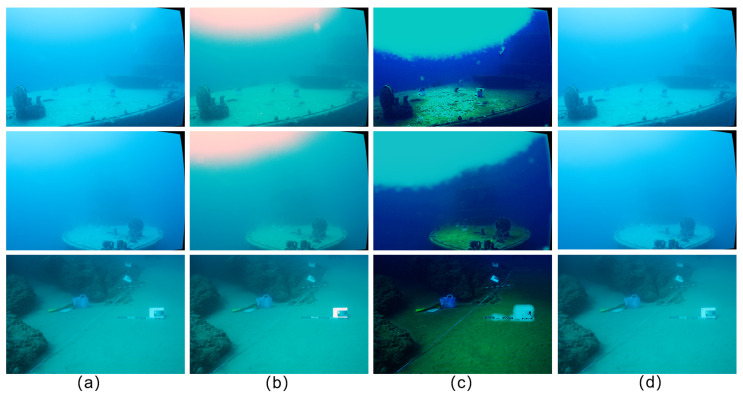
Visualization of images processed by the three modules. (**a**) Original image; (**b**) processed by Adaptive White Balance module; (**c**) processed by Red Inverse Channel Prior module; (**d**) processed by Joint Bilateral Filtering module.

**Figure 9:**
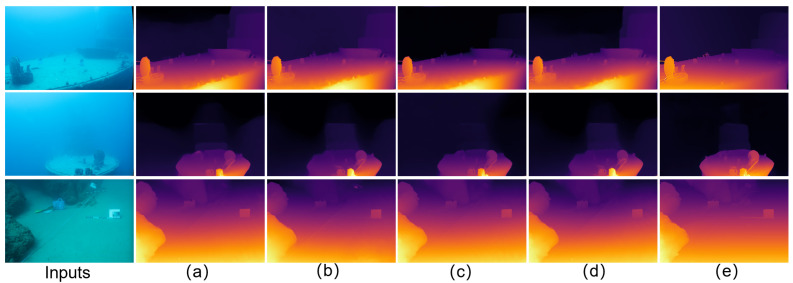
Visualization of depth maps from ablation study on the three denoising modules in dual-branch denoising: (**a**) without denoising modules; (**b**) using only Adaptive White Balance; (**c**) using Adaptive White Balance and Red Inverse Channel Prior; (**d**) using only Joint Bilateral Filtering; (**e**) full Dual-Branch Denoising.

Figure 8 and Figure 9 visualize the intermediate outputs of each module on typical shipwreck and reef scenes from SQUID [25]. The AWB module enhances image contrast and illumination balance, revealing more details in low-light regions and contributing to clearer depth recovery. The RCP module shows the most visible improvement in hazy regions: it suppresses scattering effects, mitigates haze-induced false disparity, and substantially enhances the depth consistency of distant areas. The JBF module preserves object edges and foreground structures, removing scattering noise while avoiding excessive smoothing. When combined, these modules complement each other—AWB and RCP jointly handle color correction and dehazing, while JBF refines geometric boundaries—resulting in depth maps that maintain fine details, reduced haze, and sharp edges. Notably, the largest performance boost occurs when monocular and stereo denoising are applied jointly, highlighting the advantage of the dual-branch collaborative design in achieving both detail fidelity and edge precision across challenging underwater scenes.

#### 4.6.3. Order and Branch-Misalignment Analysis

**Figure 10:**
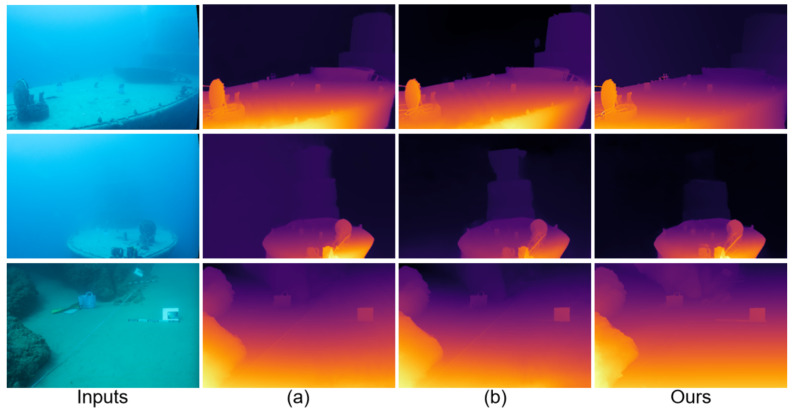
Misalignment visualization on SQUID [25]. From left to right: Input, (**a**) RCP→AWB, (**b**) Monocular JBF with Stereo AWB+RCP, JDBD (Ours).

To verify the design intention and physical consistency of our pipeline, we further conduct two misalignment tests on SQUID [25]: (i) reversing the order in the monocular pathway (RCP applied before AWB); and (ii) swapping the modules across branches (the monocular branch uses JBF, while the stereo branch uses AWB and RCP). Both variants lead to clear degradation compared with our pipeline: the reversed-order model yields EPE 1.41 and D1 3.62, and the cross-branch swap yields EPE 1.51 and D1 4.61, whereas our design (AWB→RCP for monocular; JBF for stereo) achieves EPE 1.25 and D1 2.98. See Table 7 and Figure 10.

#### 4.6.4. Rationale for the AWB→RCP Ordering

In our framework, AWB is a lightweight per-channel gain normalization computed from a high-confidence luminance subset. It expands the dynamic range of the heavily attenuated red band and stabilizes the subsequent estimation of background light $B_{\lambda ,\infty }$ and transmission t(x,λ) used by RCP. If RCP is applied first, the background-light estimation tends to be biased toward green/blue under strong spectral imbalance, which leads to residual haze or over-correction in distant regions. The misalignment results above confirm that AWB as a preconditioning step is physically consistent and beneficial for the red-inverse prior exploited by RCP.

#### 4.6.5. Ablation Takeaway

First, the three modules are complementary: AWB mainly improves illumination balance and reduces D1, RCP effectively suppresses haze in distant regions, and JBF preserves edges and photometric consistency for stereo correspondence. Second, the dual-branch collaboration (AWB+RCP for monocular and JBF for stereo) brings the largest gain, aligning with the design of mutual refinement. Third, both the reversed ordering and the cross-branch swap significantly degrade performance (1.41/3.62 and 1.51/4.61 vs. 1.25/2.98), empirically supporting the physical and algorithmic rationale of the proposed pipeline.

### 4.7. Limitations Analysis

Despite the promising results, our study still faces several limitations related to data quality and diversity.

#### 4.7.1. Quantitative Domain-Gap Assessment

We quantify the distribution shift between the synthetic UWStereo [24] domain and the real SQUID [25] domain using four indicators frequently adopted in cross-domain analysis: Fréchet Inception Distance (FID), which measures the Fréchet distance between the means and covariances of two sets in a chosen feature space; Kernel Inception Distance (KID) with a polynomial kernel, which is an unbiased estimate of the squared Maximum Mean Discrepancy (MMD); MMD with an RBF kernel defined in a reproducing-kernel Hilbert space; and UCIQE, a no-reference underwater image-quality index combining chroma dispersion, saturation, and contrast (higher generally indicates clearer water and stronger local contrast). To make the features sensitive to underwater color casts and edge statistics, FID/KID/MMD are not computed on Inception embeddings but on hand-crafted descriptors built from each image as follows: we first convert RGB to CIE Lab color space (D65 white) and take the per-pixel L, a, and b channels; we then compute a 3 × 3 Sobel gradient magnitude on the luminance channel to capture edge strength; finally, we concatenate [L, a, b, gradient-magnitude] per pixel and use these descriptors as the feature space for all three distances. This choice improves sensitivity to chromatic shifts and local contrast while keeping the computation consistent across domains. Because the feature space differs from Inception, the reported FID/KID values are intended for within-paper comparison and are not numerically interchangeable with Inception-based reports in other works. As summarized in Table 8, the cross-dataset measurements are ${\mathrm{FID}}_{\mathrm{feat}}=5.5592$, ${\mathrm{KID}}_{\mathrm{poly}}=0.0411$, and ${\mathrm{MMD}}_{\mathrm{RBF}}=0.5374$ (all lower-is-better), while the average UCIQE of SQUID exceeds that of UWStereo by +3.9106 (15.1331 vs. 11.2225; higher-is-better). Taken together, these results indicate a moderate but non-trivial shift in color, contrast, and texture statistics from synthetic to real waters, which is consistent with the observed performance drop when models trained on UWStereo are evaluated on SQUID.

#### 4.7.2. Data Quality and Calibration Errors

UWStereo [24], though large-scale, cannot fully replicate the optical complexity of real waters, leaving a residual gap to real scenes. SQUID [25] exhibits rectification inaccuracies in some stereo pairs. Parallel-baseline correction alleviates part of the error, but residual calibration noise may still influence quantitative depth evaluation. Future work should consider more precisely calibrated real datasets.

#### 4.7.3. Limited Diversity of Underwater Conditions

Existing datasets provide limited coverage of water types, turbidity levels, and depths. For example, SQUID [25] contains only 57 stereo pairs, restricting a comprehensive evaluation across diverse optical conditions. Expanding real-world datasets or building large-scale synthetic sets with more accurate physics will enable stronger validation and generalization studies.

## 5. Conclusions

In summary, this work presents a Joint Dual-Branch Denoising (JDBD) framework designed to enhance the performance of stereo depth estimation in underwater environments. The framework integrates three lightweight and complementary modules—Adaptive White Balance (AWB), Red Inverse Channel Prior (RCP), and Joint Bilateral Filtering (JBF)—which operate within the monocular and stereo pathways to jointly address color distortion, scattering, and edge degradation in underwater imagery. Comprehensive experiments on synthetic and real underwater datasets demonstrate that JDBD achieves more accurate and visually consistent depth estimation, improving both depth precision and robustness across diverse water conditions. Future work will focus on extending JDBD to more advanced dual-branch architectures and exploring domain adaptation strategies to further enhance its generalization to various underwater environments.

## Figures and Tables

**Table 1:** Comparison on a subset of the UWStereo dataset [24]. All models are evaluated under the same settings. “Ours” denotes our method. Bold values indicate the best and second-best results.

Networks	EPE ↓	D1 ↓
GwcNet [43]	6.15	41.47
SAN [46]	7.13	36.41
PsmNet [44]	5.17	24.73
COEX [45]	4.24	21.29
RAFT-Stereo [9]	2.31	8.43
Selective Stereo [10]	2.03	7.32
MonSter [7]	**1.54**	**4.21**
JDBD (Ours)	**1.02**	**2.43**

**Table 2:** Comparison of model performance on the SQUID [25] dataset. All models are validated under the same conditions. “Ours” refers to our model. Bold values indicate the best and second-best results.

Networks	EPE ↓	D1 ↓
GwcNet [43]	7.37	52.87
SAN [46]	6.14	22.32
PsmNet [44]	5.72	27.34
COEX [45]	5.26	33.81
RAFT-Stereo [9]	2.69	9.21
Selective Stereo [10]	2.76	8.41
MonSter [7]	**1.83**	**6.72**
JDBD (Ours)	**1.25**	**2.98**

**Table 3:** Comparison of depth estimation with underwater image enhancement as preprocessing on the SQUID dataset [25]. “Ours” denotes our proposed model. Bold values indicate the best and second-best results.

Network	RAFT-Stereo [9]		MonSter [7]	
	EPE ↓	D1 ↓	EPE ↓	D1 ↓
CLUIE-Net [47,48]	2.44	8.59	1.73	5.01
NU2-Net [49]	2.29	8.33	1.59	4.65
GHS-UIR [50]	**2.23**	**8.05**	**1.61**	**4.57**
HCLR-Net [51]	**2.05**	**7.37**	**1.54**	**4.05**
JDBD (Ours)	–	–	**1.25**	**2.98**

**Table 4:** Performance under increasing turbidity on the SQUID dataset [25]. Rows correspond to mild → medium → heavy. All models are evaluated under identical settings.

Turbidity	RAFT-Stereo [9]		MonSter [7]		JDBD+MonSter (Ours)	
	EPE ↓	D1 ↓	EPE ↓	D1 ↓	EPE ↓	D1 ↓
mild	2.72	9.61	1.89	6.98	1.27	3.05
medium	2.86	9.98	1.93	7.04	1.38	3.19
heavy	3.03	10.73	2.14	7.54	1.59	3.86

**Table 5:** Performance under decreasing illumination on the SQUID dataset [25]. Rows correspond to high-key → normal → low-light. All models are evaluated under identical settings.

Illumination	RAFT-Stereo [9]		MonSter [7]		JDBD+MonSter (Ours)	
	EPE ↓	D1 ↓	EPE ↓	D1 ↓	EPE ↓	D1 ↓
high-key	2.74	9.43	1.89	7.02	1.27	3.04
normal	2.95	9.74	1.94	7.34	1.36	3.17
low-light	3.01	10.02	1.99	7.78	1.41	3.35

**Table 6:** Ablation study on the three denoising modules in dual-branch denoising tested on real underwater datasets.

AWB	RCP	JBF	EPE	D1
×	×	×	1.83	6.72
✓	×	×	1.89	5.32
×	✓	×	1.54	5.43
×	×	✓	1.52	5.07
✓	✓	×	1.42	5.32
✓	×	✓	1.48	4.87
×	✓	✓	1.39	4.33
✓	✓	✓	1.25	2.98

**Table 7:** Misalignment experiments: module ordering and cross-branch swap on SQUID [25]. Our default uses AWB→RCP in the monocular branch and JBF in the stereo branch.

Variant	EPE	D1
Ours (Mono: AWB→RCP; Stereo: JBF)	1.25	2.98
Mono order reversed (RCP→AWB)	1.41	3.62
Cross-branch swap (Mono: JBF; Stereo: AWB+RCP)	1.51	4.61

**Table 8:** Quantitative domain-gap indicators between UWStereo [24] (synthetic) and SQUID [25] (real). Lower is better except UCIQE (↑). FID/KID/MMD are computed on Lab color and image-gradient features not Inception features.

Setting	${\mathrm{FID}}_{\mathrm{feat}}$ ↓	${\mathrm{KID}}_{\mathrm{poly}}$ ↓	${\mathrm{MMD}}_{\mathrm{RBF}}$ ↓	UCIQE ↑
UWStereo [24] (synthetic)	—	—	—	11.2225
SQUID [25] (real)	—	—	—	15.1331
UWStereo → SQUID	5.5592	0.0411	0.5374	+3.9106

## Data Availability

The data presented in this study are available on reasonable request from the corresponding author.

## Abbreviations

The following abbreviations are used in this manuscript:

JDBD	Joint Dual-Branch Denoising
AWB	Adaptive White Balance
RCP	Red Inverse Channel Prior
JBF	Joint Bilateral Filtering
MonSter	MonSter: Marry Monodepth to Stereo Unleashes Power
RAFT-Stereo	RAFT-Stereo: Multilevel Recurrent Field Transforms for Stereo Matching
SQUID	Stereo Quantitative Underwater Image Dataset
UWStereo	UWStereo: A Large Synthetic Dataset for Underwater Stereo Matching
